# Do intrauterine or genetic influences explain the foetal origins of chronic disease? A novel experimental method for disentangling effects

**DOI:** 10.1186/1471-2288-7-25

**Published:** 2007-06-22

**Authors:** Anita Thapar, Gordon Harold, Frances Rice, XiaoJia Ge, Jacky Boivin, Dale Hay, Marianne van den Bree, Allyson Lewis

**Affiliations:** 1 Department of Psychological Medicine, School of Medicine, Heath Park, Cardiff University, CF14 4XN, UK; 2 School of Psychology, Park Place, Cardiff University, Cardiff, UK; 3 Department of Human and Community Development, University of California, Davis, Davis, USA

## Abstract

**Background:**

There is much evidence to suggest that risk for common clinical disorders begins in foetal life. Exposure to environmental risk factors however is often not random. Many commonly used indices of prenatal adversity (e.g. maternal gestational stress, gestational diabetes, smoking in pregnancy) are influenced by maternal genes and genetically influenced maternal behaviour. As mother provides the baby with both genes and prenatal environment, associations between prenatal risk factors and offspring disease maybe attributable to true prenatal risk effects or to the "confounding" effects of genetic liability that are shared by mother and offspring. Cross-fostering designs, including those that involve embryo transfer have proved useful in animal studies. However disentangling these effects in humans poses significant problems for traditional genetic epidemiological research designs.

**Methods:**

We present a novel research strategy aimed at disentangling maternally provided pre-natal environmental and inherited genetic effects. Families of children aged 5 to 9 years born by assisted reproductive technologies, specifically homologous IVF, sperm donation, egg donation, embryo donation and gestational surrogacy were contacted through fertility clinics and mailed a package of questionnaires on health and mental health related risk factors and outcomes. Further data were obtained from antenatal records.

**Results:**

To date 741 families from 18 fertility clinics have participated. The degree of association between maternally provided prenatal risk factor and child outcome in the group of families where the woman undergoing pregnancy and offspring are genetically related (homologous IVF, sperm donation) is compared to association in the group where offspring are genetically unrelated to the woman who undergoes the pregnancy (egg donation, embryo donation, surrogacy). These comparisons can be then examined to infer the extent to which prenatal effects are genetically and environmentally mediated.

**Conclusion:**

A study based on children born by IVF treatment and who differ in genetic relatedness to the woman undergoing the pregnancy is feasible. The present report outlines a novel experimental method that permits disaggregation of maternally provided inherited genetic and post-implantation prenatal effects.

## Background

The causal risk factors and pathways leading to common clinical problems, such as cardiovascular disease, asthma, schizophrenia and depression remain largely unknown. There is consistent evidence demonstrating that inherited, genetic factors play an important role in such disorders [1,2]. Although genetic factors are of major importance, epidemiological studies show that the rates of many disorders such as cardiovascular disease, diabetes, obesity and depression have changed over time and vary geographically to an extent that is incompatible with the effects of genetic differences [3-5]. This indicates the important contribution of environmental factors. More recently, there has been growing awareness that genes and environment work together in complex ways [6,7].

One important example of this complexity is the growing evidence that exposure to many important environmental risk factors for common disorders is not random. Specifically environmental risk factors such as exposure to early adversity are not independent of an individual's genetically influenced characteristics and behaviour or those of their parents [3]. Thus, association between an environmental risk factor and a disorder could be attributable to shared inherited genetic liability that influences both the index of environmental risk and the manifestation of disorder as well as because of true environmentally mediated risk effects. As a result of this growing awareness, there has been increasing interest in using suitable research designs to investigate this issue [3]. This is important as identifying which environmental factors exert true causal environmentally mediated risk effects on complex phenotypes is an important goal for the purposes of designing prevention, risk reduction and intervention strategies.

Examples of such designs include early adoption studies of animals and humans where the postnatal environment is provided by genetically unrelated parents. These studies show that regardless of genetic liability, postnatal environmental factors have important effects on many different outcomes; for example stress susceptibility [8], renal renin-angiotensin system sensitivity [9], cognitive ability [10] and antisocial behaviour [11]

### Prenatal environmental risk factors and complex disorders

The leading causes of global disease burden are complex disorders such as cardiovascular disease and depression. There has been increasing evidence over the last twenty years that many of these disorders and health-related problems have their origins in foetal life, with early intrauterine factors hypothesised to have long term effects on health and behaviour [12,3]. This hypothesis has been supported by evidence from animal studies [13]. *In utero *programming, whereby a stimulus or insult at a sensitive period of development has lasting effects, is thought to represent a key risk pathway by bringing about long-lasting changes to the structure and metabolism of the organism [12]. Replicated links between prenatal environmental factors and chronic disease have been demonstrated: between lower birth weight and cardiovascular disease [12], diabetes [12], depression [14] and early neurocognitive problems [15]; between poor maternal nutrition during pregnancy and schizophrenia [16]; between gestational stress and anxiety/depression [17]; and between maternal smoking in pregnancy and Attention Deficit Hyperactivity Disorder (ADHD)[18]. Most of the studies testing for these associations have used cohort or case-control designs. Although longitudinal studies are an important method for identifying causal risk factors for disease and behaviour, it is often difficult to rule out the contribution of unmeasured confounders[3]. Natural experiment designs and randomised control trials that take advantage of change in exposure to a specific environmental variable are thus attractive [3]. Only a few studies have been able to test environmental risk hypotheses by using experimental interventions or natural and sometimes unfortunate change imposed on populations. Examples of such 'experiments in nature' have been those demonstrating links between poor prenatal nutrition during the Dutch [19] and Chinese famines [20] with later mental disorders, notably schizophrenia, decreased glucose tolerance [21] and coronary heart disease [22].

### Testing whether the associations between prenatal risk and disorder are the result of environmentally mediated effects or inherited genetic influences

**Figure 1:**
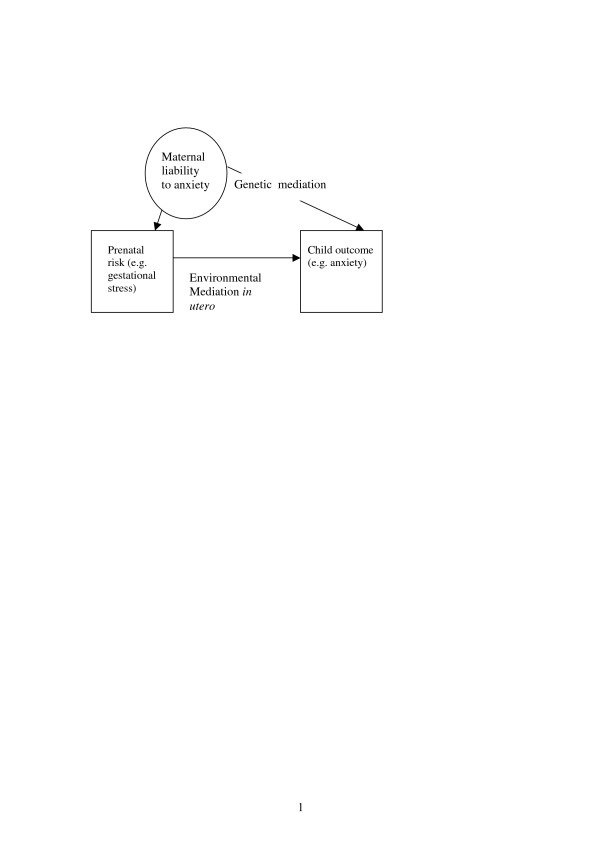
Genetic and environmental pathways between a prenatal risk factor and child outcome using the example of exposure to gestational stress and childhood anxiety.

Exposure to the maternally provided prenatal environment is not random. Many important prenatal environmental risk factors for disorder where exposure occurs *in utero*, such as gestational stress and cigarette smoking in pregnancy, are also influenced by maternal characteristics, including those that are influenced by maternal genotype [23,24]. Given this, associations between putative prenatal risk factors or indices of environmental adversity *in utero *and disease outcomes could arise through maternally provided genetic factors and/or a 'true' environmentally mediated effect (see Figure 1).

One example is the link between gestational stress and subsequent anxiety in offspring [17], where the effects are thought to be mediated by exposure to glucocorticoids *in utero*, but for which the association might be accounted for by genetic pathways, that influence both maternal predisposition to experiencing stress and offspring anxiety. Another example, is the link between pregnancy complications (such as gestational diabetes and intrauterine growth restriction) and cardiovascular disease (CVD), which may share common antecedents [25-27]. Mothers who show such complications not only have offspring who are at increased risk of showing CVD but such complications also appear to index an increased subsequent risk of vascular disease in the mothers themselves. The mechanisms for these links are not known but clearly genetic susceptibility is one potentially important contributor (see Figure 1). That is, the increased risk of cardiovascular disease in offspring may not necessarily be entirely mediated by prenatal risk effects but simply index an underlying inherited predisposition to cardiovascular disease passed on from mother to child.

Prenatal cross-fostering of animals allows disentanglement of these mechanisms and has been used in some instances to test the relative contributions of the prenatal and postnatal environment and genetic factors to different phenotypes in animals. For example, one such study demonstrated the contribution of maternally transmitted autoantibodies (i.e. prenatal environmental mediation) to diabetes in offspring. This was achieved, in part, by prenatal cross fostering non-obese diabetic mice embryos to mothers of a non-autoimmune strain [28]. The genetically susceptible mice were protected from developing diabetes by changing the maternally provided prenatal environment. Another prenatal cross fostering study found that the prenatal and postnatal maternally provided environment contributed to behavioural differences in mice [29]. More recent animal work has shown that maternally provided prenatal and postnatal environment effects on offspring may be mediated by non-inherited epigenetic mechanisms [30,31].

It should be possible to distinguish between whether maternally provided prenatal risk effects are mediated environmentally or genetically in humans by studying offspring whose intrauterine environment is provided by a genetically unrelated mother; essentially an adoption study "*in utero*". In vitro fertilisation (IVF) is becoming an increasingly common means of conception. Current estimates suggest 1.3%–3.6% of European births are now due to IVF [32]; a proportion of these births involve donated gametes and surrogacy. Children conceived via these methods may be genetically related to both parents (homologous IVF), the mother only (sperm donation), the father only (egg donation), or to neither parent (embryo donation). With gestational surrogacy, both parents are genetically related to the child but the intrauterine environment is provided by a genetically unrelated surrogate. With both egg donation and embryo donation, the mother provides the intra-uterine environment but is not genetically related to the child. Such a sample would enable maternally provided genetic and environmental effects to be separated. Such a sample could also be used to examine the contribution of genetic and environmental influences to offspring phenotypic characteristics, to complement the other designs, twin and adoption studies, already used for this purpose. In an IVF sample, this would involve examining parent/offspring phenotype resemblance, for example using correlation coefficients for continuously distributed characteristics that are calculated separately for the different conception groups and estimates of genetic and shared environmental variance estimated using the types of statistical modelling techniques used in twin studies [33]. However, given the sensitivity of artificial methods of conception, the question arises as to whether such a design is feasible and acceptable.

## Methods

Our aims were to 1) test the feasibility of identifying and recruiting a sample of children born by IVF from the 5 treatment groups, 2) establish a sample of children aged 5 to 9 years born by homologous IVF, sperm donation, egg donation, embryo donation and gestational surrogacy, 3) obtain questionnaire measures on a range of potential health and mental health related risk factors and outcomes, 4) obtain data from antenatal records for each child and 5) identify the extent to which families would be willing to engage in future research.

The questionnaire measures and antenatal data obtained were aimed at testing a range of hypotheses, specifically that associations between a) specific antenatal events (e.g. pre-eclampsia, high blood sugar), b) maternally perceived gestational stress, c) markers of prenatal-growth (e.g. birth weight, head circumference, ponderal index) and child behaviour and mental health outcomes are attributable to maternally provided environmentally mediated effects *in utero *as well as genetic factors. The research protocol was approved by Wales Multicentre Research Ethics Committee.

## Results

### Feasibility

Over the past 3 years, in an ongoing study, 18 fertility centres have participated. Families with school aged children (aged 5 to 9 years) born following IVF treatment have been recruited. To date, 741 families have participated (13 father only participated, 231 mother only participated, 497 both parents participated) although data collection is ongoing and the conception groups have been recruited in a sequential manner. The expected and current numbers in each conception group are shown in Table 1. Greater than expected numbers of homologous IVF families have been recruited. Expected numbers are based on initial power calculations and the numbers of types of IVF treatments in the UK for the age range of children. Power estimates at the start of the study using pilot data showed that power based on the expected sample sizes is sufficient to detect most effects, except those of small (defined as 0.10) or very small effect size (<0.10) [34].

**Table 1:** Current and target sample in each conception group

	Current sample size	Target sample size
Homologous IVF	378	300
Sperm donation	170	200
Embryo donation	31	50
Egg donation	146	200
Gestational Surrogacy	16	30

Among those who participated in this project, clinic staff and families reported a positive research experience, with the vast majority of families (655/741 = 88%) agreeing to receive information about future research studies. 80% (573/712) of mothers (excluding the 16 mothers from the gestational surrogacy group) agreed for researchers to access antenatal records to obtain detailed information about the pregnancy. The level of agreement between maternal report of pre-/peri-natal factors and information obtained from antenatal notes was excellent for a range of variables (e.g. birth weight (r = .991), smoking during pregnancy (kappa = .806), high blood pressure during pregnancy kappa = .716) [35]. The only exception was length of labour (kappa = .257) which was not well recalled by mothers [35]. This means that for where the focus is on prenatal variables, maternal reports alone are satisfactory in most instances. Thus the eligible sample here will include all those families where the mother has returned a questionnaire.

### Expected pattern of results for testing prenatal environmental effects

Table 2 shows the genetic relationship between the woman undergoing the pregnancy and offspring for each of the conception groups. Where an association between a maternally provided environmental risk factor (e.g. gestational diabetes) and outcome (e.g. birth weight) is environmentally and not genetically mediated, association would be observed in families where the woman undergoing pregnancy and offspring are genetically related (homologous IVF, sperm donation) and also found to be significant in the offspring who are genetically unrelated to the woman who undergoes the pregnancy (egg donation, embryo donation, surrogacy). Where an association between the risk factor and outcome is entirely genetically mediated, we would expect to observe an association in families where the woman undergoing pregnancy and offspring are genetically related but not in those who are unrelated. Where genetic and environmental mediation both contribute, association will be observed in genetically related and unrelated dyads. The expected pattern of results for each of these scenarios is shown in table 2. Thus, for example, if prenatal stress effects on offspring anxiety symptoms were entirely genetically mediated, we would expect association between these variables in the homologous IVF and sperm donation groups but not in the other groups. Associations for continuously distributed variables will be examined using regression analysis. Relevant covariates such as being a twin will need to be included but by necessity will differ depending on the outcome of interest. Differences in the degree of association between risk factor and outcome for the groups where the dyads are genetically related (homologous IVF, sperm donation) vs. those who are genetically unrelated will be assessed.

**Table 2:** Genetic relationship between the woman who undergoes the pregnancy and offspring in each conception group and predicted patterns of association between prenatal risk factor X and outcome Y for a) environmentally mediated and b) genetically mediated effects.

		Association between X and Y	
	Genetic relationship Between pregnant woman and offspring	Environmental Mediation	Genetic Mediation
Homologous IVF	Yes	yes	yes
Sperm donation	Yes	yes	yes
Embryo donation	No	yes	no
Egg donation	No	yes	no
Gestational Surrogacy	No	yes	no

### Antenatal and peri-natal risk factors in the sample

Table 3 shows rates of a number of maternally reported antenatal and peri-natal complications and intrauterine risk exposures in this sample. Table 4 shows maternal and paternal age at birth of the child for the sample so far. The mean age of the children was 6.76 years. There were 380 (51.3%) boys and 361 (48.7%) girls. The vast majority of children lived with their mother and father (679; 91.6%), 45 (6.0%) lived with their mother only, 8 (1.1% lived with their mother and step-father) and 9 children had other living arrangements such as shared residency, lived with father only or with lesbian parents. As expected, there is evidence of elevated rates of certain types of complications during pregnancy. For instance, hypertensive disorders are estimated to occur in up to 10% of all pregnancies [36] thus the rate of nearly 15% in the present sample is above expected levels although this may be attributable to older maternal age. Approximately 8% of infants in the UK are born weighing less than 2500 grams [39]. The elevated rate in the present sample seems to be due to the high proportion of multiple births (22.7% of the sample) given that the prevalence of low birth weight is 8.6% when multiple births are excluded. Conversely, rates of maternal smoking during pregnancy are lower than general U.K. population estimates [18]. This pattern of results is consistent with other studies across the world which show that the rate of certain, but not all, antenatal and peri-natal complications is increased in IVF versus naturally conceived pregnancies but that this is mainly attributable to high rates of multiple births and increased maternal age and that outcomes are more favourable in single embryo transfers [38]. Table 4 illustrates increased maternal and paternal age compared to the UK average [39]. There has been no evidence of increased rates of psychiatric disorder or symptoms found in this study or in previous published studies [40]. In fact, the children in this sample showed psychological adjustment levels (mean emotional and behavioural symptom scores) very similar to normative data for the UK [41]. For example, the mean conduct problem score assessed by the Strengths and Difficulties Questionnaire [42] was 1.50, standard deviation = 1.42 and normative data for UK children aged 5 to 10 years shows a mean of 1.6 and a standard deviation of 1.7.

**Table 3:** Examples of rates of exposure to pre and peri-natal risk factors in all conception groups

	Homologous IVF N (%)	Sperm donation N (%)	Egg donation N (%)	Embryo donation N (%)	Gestational surrogacy N (%)	Total N (%)
High blood pressure during pregnancy requiring hospitalisation	40 (10.8%)	22 (13.1%)	37 (26.1%)	5 (17.2%)	1 (6.7%)	105 (14.5%)
High blood sugar during pregnancy	21 (6.0%)	10 (6.0%)	15 (10.7%)	0 --	2 (13.3%)	48 (6.8%)
Cigarette smoking during pregnancy	25 (6.8%)	7 (4.2%)	4 (2.8%)	3 (10.3%)	1 (7.1%)	40 (5.5%)
Alcohol during pregnancy	89 (24.1%)	46 (27.4%)	34 (23.8%)	3 (10%)	3 (21.4%)	175 (24.1%)
Multiple birth	76 (20.1%)	45 (24.1%)	35 (24.1%)	6 (19.4%)	6 (37.5%)	168 (22.7%)
Low infant birth weight ^a^	55 (14.7%)	33 (19.6%)	34 (23.4%)	5 (16.7%)	6 (40%)	133 (18.2%)
Low infant birth weight ^a ^(excluding multiple births)	20 (6.7%)	10 (8.0%)	15 (13.6%)	3 (12.5%)	1 (11.1%)	49 (8.6%)

^a ^low birth weight is defined as 2500 grams or less

-- percentage not calculable

**Table 4:** Maternal and paternal age at child birth for all conception groups

	Homologous IVF	Sperm donation	Egg donation	Embryo donation	Gestational surrogacy	Total
	Mean SD Range	Mean SD Range	Mean SD Range	Mean SD Range	Mean SD Range	Mean SD Range
Maternal age at birth of child	34.14	33.88	37.88	41.23	36.07	35.15
	3.53	3.82	5.89	6.21	5.34	4.74
	25, 45	25, 43	23, 55	30, 54	27, 47	23, 55
Paternal age at birth of child	36.84	38.73	38.78	45.61	38.00	38.01
	5.84	7.02	6.64	7.02	6.47	6.57
	23, 71	27, 60	24, 58	34, 62	27, 53	23, 71

## Discussion

These data show that this novel design is feasible and can be successfully employed in the U.K. to test the effects of maternally provided prenatal environment on offspring. As with all other research designs there are strengths and weakness. Issues that will need consideration are the effects of programming at the pre-implantation stage, the impact of differences in IVF methods and statistical power in relation to group and sub-group comparisons. Potential limitations include under-representation of some risk factors as well as health problems related to older maternal age and IVF. The objective of this design is not to obtain an epidemiologically representative sample but rather to test differences in the degree of association between risk factors and outcome across different conception groups. Thus it is important to consider whether specific associations differ in strength between the homologous IVF group and the general population. Given the current numbers of children born by IVF in the rare groups, this design will be most useful for examining trait measures or common conditions and risk factors such as emotional and behavioural symptoms, cognitive ability, asthma, blood pressure and BMI. For many outcomes, both clinical conditions and traits that appear later in life, the children will need to be followed up into adult life.

Retrospectively obtaining information on pregnancy complications for the surrogacy group was difficult because commissioning parents were often not aware of details of the pregnancy and families were either no longer in touch with the surrogate or did not wish her to be contacted. Thus information was difficult to obtain from the surrogate who experienced the pregnancy. However, this is not crucial for the design as the egg donation and embryo donation groups also allows the researcher to separate the effects of genetic transmission and prenatal environmental exposure and here, pregnancy data are easily available.

In summary there is evidence that many health problems have their origins in foetal life and are also genetically influenced. Associations between prenatal adversity and many health outcomes arise because of either true environmentally mediated effects or inherited genetic factors or both. There is a need for experimental research designs that enable disentanglement of inherited genetic from environmentally mediated risk effects. To date twin and adoption study designs of clinical disorders and behaviours have been used to separate the effects of postnatal and later environmental influences from inherited factors [3]. However such traditional designs cannot separate maternally provided prenatal environmental risk effects from inherited genetic influences on outcomes. Thus new, genetically sensitive experimental designs are needed. In addition to the novel design we propose, it is also possible to use a design whereby the offspring of adult twins are examined. Here, the offspring of monozygotic ("identical") twin pairs will be social cousins but biological half siblings (sharing on average 25% of their inherited genes). This design has different strengths and weaknesses from the new method proposed here [37]. For instance, for genetically influenced maternal behaviours, levels of discordant risk exposure to prenatal adversity will be expected to be low for the offspring of adult MZ twins. However, the offspring of adult twins design has been used to illustrate that the association between maternal smoking during pregnancy and low birth weight is environmentally rather than genetically mediated [37] consistent with an intrauterine effect. Both research designs provide complimentary approaches to disentangling the pathways involved in the aetiology of disease.

## Conclusion

In conclusion, there is increasing evidence that prenatal risk factors may contribute to the aetiology of different disorders and traits. The observed associations between prenatal risk factors and specific outcomes could arise through genetic pathways. We present a novel method that appears to be feasible for testing whether associations between putative prenatal risk factors and different outcomes are attributable to an environmentally mediated effect.

## Competing interests

AT has received educational, non promotional grants from pharmaceutical companies. The other authors declare that they have no competing interests.

## Authors' contributions

AT and GH jointly contributed to the idea and scientific research design. XJG also contributed to the idea. FR has undertaken project management and supervision and generated the data. JB provided links to Fertility Centres, with JB and FR adding additional expertise relating to specific IVF subgroup considerations. DH and MVB are co-applicants on the grant and will be involved in analysis and measurement. All authors contributed to the writing and editing.

## Pre-publication history

The pre-publication history for this paper can be accessed here:



## References

[B1] McGuffin P, Riley B, Plomin R (2001). Genomics and behavior. Toward behavioral genomics. Science.

[B2] Subramanian G, Adams MD, Venter JC, Broder S (2001). Implications of the human genome for understanding human biology and medicine. JAMA.

[B3] Rutter M, Pickles A, Murray R, Eaves L (2001). Testing hypotheses on specific environmental causal effects on behavior. Psychol Bull.

[B4] Murray CJL, Lopez AD (1997). Alternative projections of mortality and disability by cause 1990–2020: Global Burden of Disease Study. Lancet.

[B5] Prentice AM, Jebb SA (1995). Obesity in Britain – Gluttony or Sloth. BMJ.

[B6] Rutter M (2006). Genes and behavior: Nature-nurture interplay explained.

[B7] Institute of Medicine Board on Health Sciences Policy (2006). Genes, behavior and the social environment: moving beyond the nature nurture debate.

[B8] Prakash P, Merali Z, Kolajova M, Tannenbaum BM, Anisman H Maternal factors and monoamine changes in stress-resilient and susceptiblemice: cross-fostering effects. Brain Res.

[B9] Gouldsborough I, Lindop GB, Ashton N (2003). Renal renin-angiotensin system activity in naturally reared and cross-fostered spontaneously hypertensive rats. Am J Hypertens.

[B10] Schiff M, Duyme M, Dumaret A, Stewart J, Tomkiewicz S, Feingold J Intellectual status of working-class children adopted early into upper-middle-class families. Science.

[B11] Cadoret RJ, Cain CA, Crowe RR (1983). Evidence for gene-environment interaction in the development of adolescent antisocial behavior.

[B12] Barker D (1998). Mothers, babies and health in later life.

[B13] Seckl JR, Meaney CJL (2004). Glucocorticoid programming. Ann N Y Academic Sci.

[B14] Gale CR, Martyn CN (2004). Birth weight and later risk of depression in a national birth cohort. B J Psychiatry.

[B15] Bhutta AT, Cleves MA, Casey PH, Cradock MM, Anand KJS (2002). Cognitive and behavioral outcomes of school-aged children who were born preterm: a meta-analysis. JAMA.

[B16] Neugebauer R (2005). Accumulating Evidence for Prenatal Nutritional Origins of Mental Disorders. JAMA.

[B17] Glover V, O'Connor TG (2002). Effects of antenatal stress and anxiety:Implications for development and psychiatry. B J Psychiatry.

[B18] Langley K, Rice F, van den Bree MB, Thapar A (2005). Maternal smoking during pregnancy as an environmental risk factor for attention deficit hyperactivity disorder behaviour: A review. Minerva Pediatrica.

[B19] Susser E, Neugebauer R, Hoek HW, Brown AS, Lin S, Labovitz D, Gorman JM (1996). Schizophrenia after prenatal famine: further evidence. Arch Gen Psychiatry.

[B20] St Clair D, Xu M, Wang P, Yu Y, Fang Y, Zhang F, Zheng X, Gu N, Feng G, Sham P, He L (2005). Rates of adult schizophrenia following prenatal exposure to the Chinese Famine of 1959–1961. JAMA.

[B21] Ravelli AC, van der Meulen JH, Michels RP, Osmond C, Barker DJ, Hales CN, Bleker OP Glucose tolerance in adults after prenatal exposure to famine. Lancet.

[B22] Roseboom TJ, van der Meulen JH, Osmond C, Barker DJ, Ravelli AC, Schroeder-Tanka JM, van Montfrans GA, Michels RP, Bleker OP (2000). Coronary heart disease after prenatal exposure to the Dutch famine, 1944–45. Heart.

[B23] Vaessen N, Janssen JA, Heutink P, Hofman A, Lamberts SWJ, Oostra BA, Pols HA, Van Duijn CM (2002). Association between genetic variation in the gene for insulin-like growth factor-I and low birthweight. Lancet.

[B24] Madden PAF, Pedersen NL, Kaprio J, Koskenvuo MJ, Martin NG (2004). The epidemiology and genetics of smoking initiation and persistence: Crosscultural comparisons of twin study results. Twin Research.

[B25] Csorba TR, Edwards AL (1995). The genetics and pathophysiology of type II and gestational diabetes. Crit Rev Clin Lab Sci.

[B26] Sattar N (2004). Do pregnancy complications and CVD share common antecedents?. Atherosclerosis Supplements.

[B27] Ray JG, Vermeulen MJ, Schull MJ, Redelmeier DA (2005). Cardiovascular health after maternal placental syndromes (CHAMPS): population-based retrospective cohort study. Lancet.

[B28] Greeley SA, Katsumata M, Yu L, Eisenbarth GS, Moore DJ, Goodarzi H, Barker CF, Naji A, Noorchashm H (2002). Elimination of maternally transmitted autoantibodies prevents diabetes in nonobese diabetic mice. Nat Med.

[B29] Francis DD, Szegda K, Campbell G, Martin WD, Insel TR (2003). Epigenetic sources of behavioral differences in mice. Nat Neurosci.

[B30] Meaney MJ, Szyf M (2005). Environmental programming of stress responses through DNA methylation: life at the interface between a dynamic environment and a fixed genome. Dialogues Clin Neurosci.

[B31] Gluckman PD, Hanson MA, Beedle AS (2007). Non-genomic transgenerational inheritance of disease risk. Bioessays.

[B32] Nygren KG, Nyboe Andersen A (1999). Assisted reproductive technology in Europe, 1999: Results generated from European registers by ESHRE. Human Reproduction.

[B33] Kendler KS, Prescott CA (2006). Genes, Environment and Psychopathology: Understanding the Causes of Psychiatric and Substance Use Disorders.

[B34] Cohen J (1988). Statistical power analysis for the behavioral sciences.

[B35] Rice F, Lewis A, Harold G, van den Bree M, Boivin J, Hay DF, Owen MJ, Thapar A Agreement between maternal report and antenatal records for a range of pre and peri-natal factors: The influence of maternal and child characteristics. Early Hum Dev.

[B36] American College of Obstetricians and Gynecologists (2001). ACOG practice bulletin. Clinical management guidelines for obstetrician-gynecologists. Number 29: chronic hypertension in pregnancy. Obstetrics & Gynecology.

[B37] D'Onofrio BM, Turkheimer EN, Eaves LJ, Corey LA, Berg K, Solaas MH, Emery RE (2003). The role of the children of twins design in elucidating causal relations between parent characteristics and child outcomes. J Child Psychol Psychiatry.

[B38] De Neubourg D, Gerris J, Mangelschots K, Van Royen E, Vercruyssen M, Steylemans A, Elseviers M (2006). The obstetrical and neonatal outcome of babies born after single-embryo transfer in IVF/ICSI compares favourably to spontaneously conceived babies. Hum Reprod.

[B39] Birth statistics (2005). Review of the registrar general on births and patterns of family building in England and Wales.

[B40] Golombok S, Brewaeys A, Giavazzi MT (2002). The European study of assisted reproduction families: the transition to adolescence. Hum Reprod.

[B41] Meltzer H, Gatwood R, Goodman R, Ford T (2000). The Mental Health and Well being of Children and Adolescents in Great Britain.

[B42] Goodman R (1997). The Strengths and Difficulties Questionnaire: a research note. J Child Psychol Psychiatry.

